# Thickness dependency of field emission in amorphous and nanostructured carbon thin films

**DOI:** 10.1186/1556-276X-7-286

**Published:** 2012-06-01

**Authors:** Maziar Shakerzadeh, Edwin Hang Tong Teo, Beng Kang Tay

**Affiliations:** 1 School of Electrical and Electronic Engineering, Nanyang Technological University, 50 Nanyang Avenue, Singapore, 639798, Singapore; 2 Temasek Laboratories, Nanyang Technological University, 50 Nanyang Avenue, Singapore, 639798, Singapore; 3 CINTRA, CNRS/NTU/THALES, UMI 3288, Research Techno Plaza, 50 Nanyang Drive, Border X Block, Level 6, Singapore, 637553, Singapore

**Keywords:** Carbon films, Preferred orientation, Field emission

## Abstract

Thickness dependency of the field emission of amorphous and nanostructured carbon thin films has been studied. It is found that in amorphous and carbon films with nanometer-sized *sp*^2^ clusters, the emission does not depend on the film thickness. This further proves that the emission happens from the surface *sp*^2^ sites due to large enhancement of electric field on these sites. However, in the case of carbon films with nanocrystals of preferred orientation, the emission strongly depends on the film thickness. *sp*^2^-bonded nanocrystals have higher aspect ratio in thicker films which in turn results in higher field enhancement and hence easier electron emission.

## Background

Field emission (FE) from amorphous and nanostructured carbon thin films is widely studied in the past few years [1-7]. Understanding the emission mechanism and reducing the threshold field at which the emission occurs (*F*_th_) are the main two subjects of interest. Amorphous carbon (a-C) films are formed from carbon atoms of *sp*^2^ and *sp*^3^ hybridization [8]. Mechanical and physical properties of a-C films strongly depend on the *sp*^2^ percentage as well as the presence and size of *sp*^2^ (sub) nanoclusters embedded in *sp*^3^ matrix.

Different field enhancement mechanisms have been proposed for easy electron emission from carbon thin films. Robertson found that for different types of carbon films (hydrogenated, nitrogenated, etc.), there is an optimum *sp*^2^ cluster size at which the emission occurs at the lowest possible *F*_th_[8]. Based on this observation, Carey et al. [2] have proposed that the large field enhancement of carbon films is mainly due to the presence and distribution of conductive *sp*^2^ nanoclusters embedded in insulative *sp*^3^ matrix. The presence of a conductive sphere embedded in an insulating matrix leads to small field enhancement. However, the presence of two or more such spheres can further increase the enhancement; for instance, the field enhancement of two conductive spheres in the bispherical coordination system was studied, and it was found that the presence of two gold spheres which are placed 5 nm apart from each other results in enhancements of 56 which can be increased to 400 for a 1-nm separation [9]. Based on this theory, the emission only occurs from the surface clusters and hence is independent of the thickness.

Despite the above-mentioned theory, Forrest et al. [3] studied the effect of different parameters including film thickness on FE of pure, nitrogenated, and hydrogenated a-C thin films. They found an optimum thickness for the lowest *F*_th_ in hydrogenated and nitrogenated carbon films. Since there is no correlation between the surface microstructure and the film thickness, this finding is obviously in contradiction with the mechanism proposed by Carey et al. [2]. The only mechanism which can be used to describe the thickness dependency of FE is space-charge interlayer-induced band bending for semiconductors [10]. Carrier depletion across the film thickness results in field enhancement at the Si/C interface; therefore, the electrons will be emitted from the conduction band of the silicon substrate to highly curved conduction band of the film. At very thin samples, although emitted electrons possess very high energies, they still cannot overcome the emission barrier (the work function of the film). At very thick films, on the other hand, the electrons will lose energy while they are passing the film, and hence they cannot overcome the emission barrier. Therefore, there is an optimum film thickness at which the emission occurs at the lowest possible field.

In another study, Zhao et al. [11] studied the thickness dependency of FE of a-C films. It was found that for a pure a-C film deposited at 200-V substrate bias, the *F*_th_ does not strongly depend on the film thickness. By challenging the space-charge interlayer-induced band bending model proposed by Forrest, they suggest that the F-N tunneling theory is the most suitable model to describe the emission from a-C films.

More recently, the formation of preferred orientation [12-15] and the effect of this texture on properties of carbon films [16-18] attract lots of theoretical and experimental attentions. In our previous work, we have shown that the formation of preferred orientation in the microstructure of the film results in an abrupt decrease in the *F*_th_. It was discussed that the formation of conductive *sp*^2^ channels throughout the thickness of the film results in the formation of high-aspect-ratio filaments which enhances the local field significantly.

In this paper, in order to reconfirm the mechanisms mentioned above, the thickness dependency of FE in amorphous (with different bonding structures) carbon films with *sp*^2^-bonded (sub) nanocrystals has been studied. Besides, the thickness dependency of carbon films with nanocrystals of preferred orientation has been studied.

## Methods

Filtered cathodic vacuum arc [19] was used to prepare different types of amorphous and nanocrystalline carbon films. Bonding structure of the films was controlled through controlling the negative substrate bias during the deposition. In order to fabricate carbon thin films with nanocrystals of preferred orientation, a carbon film deposited at 300-V substrate bias was irradiated by a single pulse of a 248-nm excimer laser with a pulse width of 23 ns. The laser energy was kept at 460 mJ/cm^2^. FE was tested in a parallel plate configuration with an indium tin oxide-coated glass as the cathode with an anode-cathode spacing of 100 μm in a pressure lower than 5 × 10^−6^ Torr. In order to check the repeatability of the data, two samples were prepared at each condition. FE tests have been done on two different positions of every individual sample. More than ten measurements have been done on each test spot.

## Results and discussion

**Figure 1:**
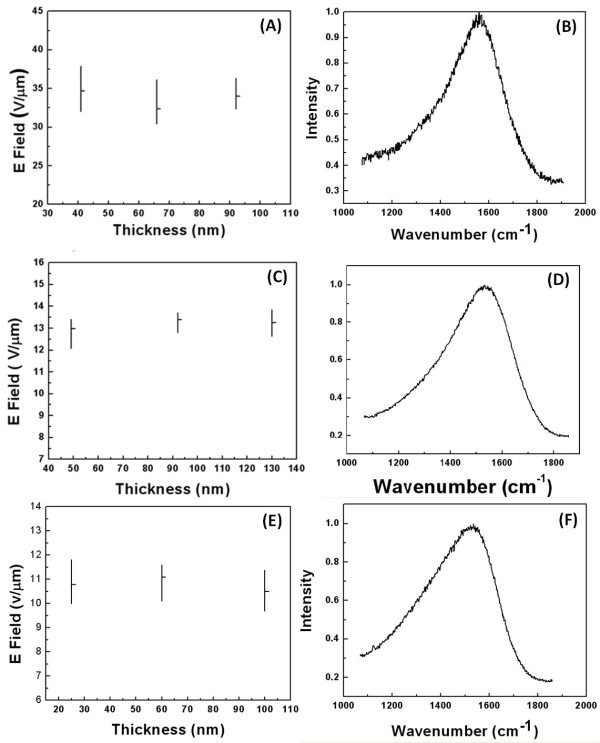
**Thickness dependency of field emission and Raman spectra of carbon films.** Deposited at (**A**, **B**) 100, (**C**, **D**) 1,000, and (**E**, **F**) 2,000 V.

Figure 1 shows the Raman spectra and thickness dependency of field emission from three different a-C films. The first film (Figure 1A,B) is a tetrahedral amorphous carbon film deposited at 100-V substrate film. As it can be deduced from the Raman spectra (Figure 1B) which can be fitted by a single Breit-Wigner-Fano (BWF) curve centered at 1,560 cm^−1^, the *sp*^3^ content of the film can be estimated to be about 75%. Moreover, there are no *sp*^2^ clusters formed in the microstructure as no D band is required to fit the spectra [20]. Therefore, the film can be considered as a pure amorphous film. As it is shown in Figure 1A, there is no distinct relation between the film thickness and emission *F*_th_, and the average *F*_th_ for all thicknesses is about 32 to 35 V/μm. Very high *F*_th_ can be understood by considering the high *sp*^3^ content of the films. Increasing the substrate bias to 1,000 V increases the *sp*^2^ content of the film to 60% (Figure 1D). However, since the Raman spectra can still be fitted by a single BWF curve centered at 1,531 cm^−1^, no clustering of *sp*^2^ bonded atoms is formed in the microstructure. As it is shown in Figure 1C, the emission from this film is still independent of the thickness. However, it is noticeable that compared to the film deposited at the 100-V bias, an increase in the *sp*^2^ content of the film decreases the *F*_th_ dramatically. The average *F*_th_ in 1,000-V deposited films is found to be 13 V/μm. However, increasing the deposition bias to 2,000 V results in the formation of *sp*^2^ clusters embedded in the amorphous matrix with an in-plane cluster size of about 1 nm in diameter. The Raman spectra can be fitted by a G band centered at 1,542 cm^−1^ and a D band with an *I*_D_/*I*_G_ of 0.35. The in-plane *sp*^2^ cluster size can be estimated considering the *I*_D_/*I*_G_:

(1) $$\frac{I_{D}}{I_{G}}=C_{\lambda }^{/}\;L_{a}^{2}$$

where $C_{\lambda }^{/}$ is 0.0055 for the 514-nm laser which was used in this study. As it can be observed, similar to previous purely amorphous films, there is still no correlation between the film thickness and *F*_th_. Moreover, the presence of *sp*^2^ nanoclusters results in the reduction of average *F*_th_ to 10 to 11 V/μm.

**Figure 2:**
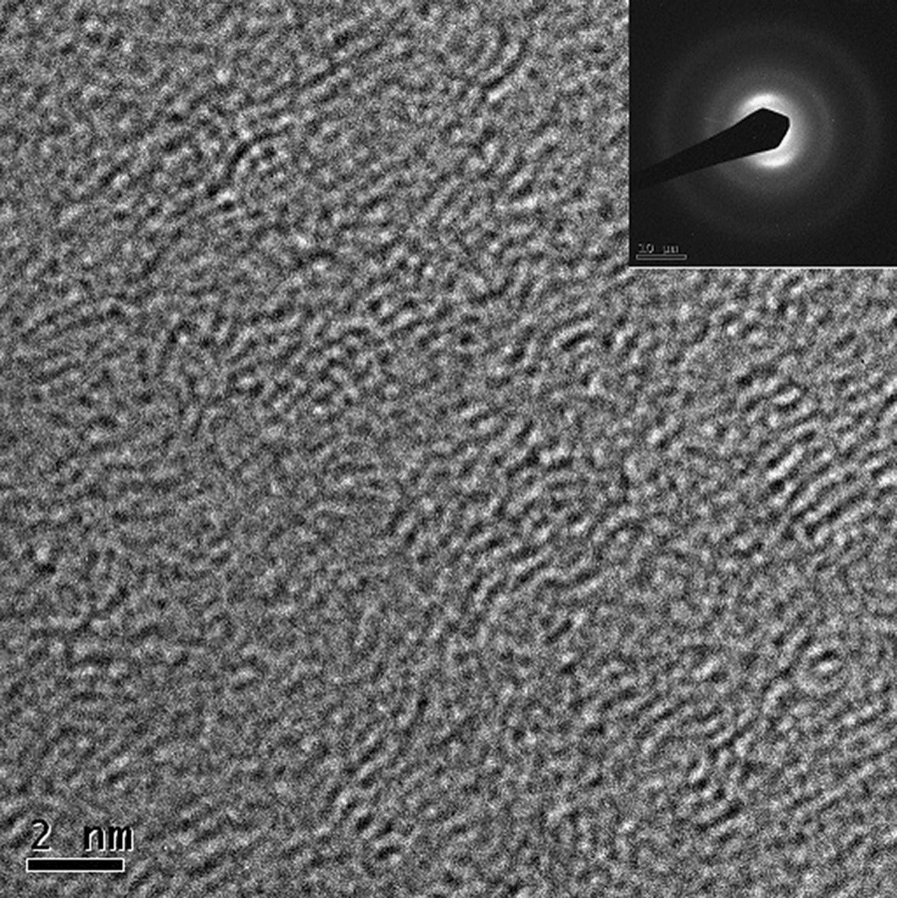
**HRTEM images and the respective diffraction pattern (inset) of carbon films.** Deposited at 300 V and irradiated by a 462.5-mJ/cm^2^ laser.

Figure 2 shows the high-resolution transmission electron microscope (HRTEM) image of the carbon film deposited at 300-V substrate bias and irradiated by a single pulse of 460-mJ/cm^2^ excimer laser. As it can be observed from the HRTEM image and the respective diffraction pattern, (002) graphitic basal planes are oriented perpendicular to the substrate. The formation and propagation of preferred orientation in the microstructure of the carbon films can be explained using the high internal stress and also the anisotropic nature of graphite crystal structure. McKenzie and Bilek [12] have shown that during the graphitization of a-C films, the system possesses its lowest free energy (and hence the most stable phase) when the basal planes are oriented in the direction perpendicular to the stress plane (in this case, the growth plane).

**Figure 3:**
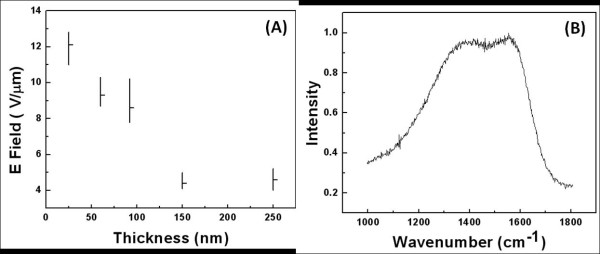
**Thickness dependency of field emission and respective Raman spectra.** (**A**) Thickness dependency of field emission of carbon film irradiated by a single pulse of 460 mJ/cm^2^ and (**B**) respective Raman spectra of the film.

Figure 3 shows the evolution of *F*_th_ of the 460-mJ/cm^2^ single pulse laser-irradiated carbon film as a function of film thickness. Despite the amorphous films, there is a direct relation between the film thickness and the *F*_th_. Films of 25-nm thick show relatively high *F*_th_ of about 12 V/μm. Increasing the film thickness to 150 nm decreases the *F*_th_ to about 4 V/μm. Further increase in film thickness (250 nm) does not alter the emission threshold significantly. High-energy laser irradiation results in the formation of conductive channels throughout the thickness of the film which decreases the *F*_th_ through the formation of high-aspect-ratio filaments. Increasing the film thickness increases the aspect ratio of the filaments, and as it is shown in Figure 3A, the *F*_th_ decreases. The film shows its lowest *F*_th_ at about 150 nm. However, further increase in film thickness does not affect the *F*_th_ noticeably. This can be due to the fact that although the film thickness is increased, the average filament length and hence the aspect ratio is kept constant. This, in turn, is due to the non-homogeneity and non-continuity of conductive filaments throughout the thickness of the film.

## Conclusions

Thickness dependency of the field emission of amorphous and nanostructured carbon thin films has been studied in this work. It was found that regardless of the bonding structure and clustering of *sp*^2^-bonded atoms, emission threshold field is independent of the film thickness. However, the field emission from carbon films with nanocrystals of preferred orientation strongly depends on the film thickness. Increasing the film thickness results in the increase in the aspect ratio of conductive *sp*^2^-bonded filaments and hence decreases the threshold field through increasing the enhancement factor.

## Competing interests

The authors declare that they have no competing interests.

## Authors’ contributions

MS and EHTT contributed on performing the experiments and writing the paper. Professor TBK contributed as the scientific advisor of the project upon performing the experiments. All authors read and approved the final manuscript.

## Authors’ information

MS is currently working as a research scientist in the Data Storage Institute (DSI). He is studying the mechanical electrical and thermal properties of carbon thin films and nanostructures. EHTT is working as NTU-DSO postdoc fellow studying electrical mechanical and thermal properties of nanomaterials. TBK is a professor in the School of Electrical and Electronic Engineering, NTU. His team is working on the synthesis, characterization, and applications of nanomaterials and thin films.

## References

[B1] Carey JD Forrest RD Khan RUA Silva SRP Influence of sp2 clusters on the field emission properties of amorphous carbon thin films Appl Phys Lett 2000 77 2006 2008 10.1063/1.1312202

[B2] Carey JD Forrest RD Silva SRP Origin of electric field enhancement in field emission from amorphous carbon thin films Appl Phys Lett 2001 78 2339 2341 10.1063/1.1366369

[B3] Forrest RD Burden AP Silva SRP Cheah LK Shi X A study of electron field emission as a function of film thickness from amorphous carbon films Appl Phys Lett 1998 73 3784 3786 10.1063/1.122894

[B4] Hart A Satyanarayana BS Milne WI Robertson J Field emission from tetrahedral amorphous carbon as a function of surface treatment and substrate material Appl Phys Lett 1999 74 1594 10.1063/1.123627

[B5] Ilie A Ferrari AC Yagi T Robertson J Effect of sp2-phase nanostructure on field emission from amorphous carbons Appl Phys Lett 2000 76 2627 10.1063/1.126430

[B6] Ilie A Hart A Flewitt AJ Robertson J Milne WI Effect of work function and surface microstructure on field emission of tetrahedral amorphous carbon J Appl Phys 2000 88 6002 6010 10.1063/1.1314874

[B7] Satyanarayana BS Hart A Milne WI Robertson J Field emission from tetrahedral amorphous carbon Appl Phys Lett 1997 71 1430 10.1063/1.119915

[B8] Robertson J Diamond-like amorphous carbon Materials Science and Engineering: R: Reports 2002 37 129 281 10.1016/S0927-796X(02)00005-0

[B9] Chaumet PC Dufour JP Electric potential and field between two different spheres J Electrost 1998 43 145 159 10.1016/S0304-3886(97)00170-8

[B10] Amaratunga GAJ Silva SRP Nitrogen containing hydrogenated amorphous carbon for thin-film field emission cathodes Applied Physics Letters 1996 68 2529 2531 10.1063/1.116173

[B11] Zhao JP Chen ZY Wang X Shi TS Yano T Thickness-independent electron field emission from tetrahedral amorphous carbon films Appl Phys Lett 2000 76 191 193 10.1063/1.125699

[B12] McKenzie DR Bilek MMM Thermodynamic theory for preferred orientation in materials prepared by energetic condensation Thin Solid Films 2001 382 280 287 10.1016/S0040-6090(00)01702-8

[B13] Shakerzadeh M Teo EHT Sorkin A Bosman M Tay BK Su H Plasma density induced formation of nanocrystals in physical vapor deposited carbon films Carbon 2011 49 1733 1744 10.1016/j.carbon.2010.12.059

[B14] Lau DWM Moafi A Taylor MB Partridge JG McCulloch DG Powles RC McKenzie DR The structural phases of non-crystalline carbon prepared by physical vapour deposition Carbon 2009 47 3263 3270 10.1016/j.carbon.2009.07.044

[B15] Teo EHT Bolker A Kalish R Saguy C Nano-patterning of through-film conductivity in anisotropic amorphous carbon induced using conductive atomic force microscopy Carbon 2011 49 2679 2682 10.1016/j.carbon.2011.02.055

[B16] Shakerzadeh M Xu N Bosman M Tay BK Wang X Teo EHT Zheng H Yu H Field emission enhancement and microstructural changes of carbon films by single pulse laser irradiation Carbon 2011 49 1018 1024 10.1016/j.carbon.2010.11.010

[B17] Tan CW Maziar S Teo EHT Tay BK Microstructure and through-film electrical characteristics of vertically aligned amorphous carbon films Diamond Relat Mater 2011 20 290 293 10.1016/j.diamond.2011.01.010

[B18] Shakerzadeh M Samani MK Khosravian N Teo EHT Bosman M Tay BK Thermal conductivity of nanocrystalline carbon films studied by pulsed photothermal reflectance Carbon 2012 50 1428 1431 10.1016/j.carbon.2011.10.015

[B19] Tay BK Zhao ZW Chua DHC Review of metal oxide films deposited by filtered cathodic vacuum arc technique Materials Science and Engineering: R: Reports 2006 52 1 48 10.1016/j.mser.2006.04.003

[B20] Ferrari AC Libassi A Tanner BK Stolojan V Yuan J Brown LM Ridil SE Kleinsorge B Robertson J Density, sp3 fraction, and cross-sectional structure of amorphous carbon films determined by X-ray reflectivity and electron energy-loss spectroscopy Phys Rev B: Condens Matter 2000 62 11089 11103 10.1103/PhysRevB.62.11089

